# Surgical technique and implant design affect abduction kinematics and functional outcomes after reverse shoulder arthroplasty

**DOI:** 10.1016/j.medengphy.2025.104323

**Published:** 2025-03-10

**Authors:** Gillian Kane, Clarissa LeVasseur, Ajinkya Rai, Maria Munsch, Alexandra S. Gabrielli, Christopher J. Como, Jonathan D. Hughes, William Anderst, Albert Lin

**Affiliations:** a Department of Orthopaedic Surgery, University of Pittsburgh, Pittsburgh, PA, USA; b School of Medicine, University of Pittsburgh, Pittsburgh, PA, USA

**Keywords:** Shoulder, Strength, Kinematics, Arthrokinematics, Biplane radiography

## Abstract

The purpose of this study was to identify surgical techniques and implant geometries that influence *in-vivo* kinematics, functional outcomes, and clinical outcomes after reverse shoulder arthroplasty (RSA). Synchronized biplane radiographs imaged the operated shoulder during scapular plane abduction in 35 patients who received RSA within the past 2.5 ± 1.2 yrs. Shoulder kinematics and arthrokinematics (contact paths) were determined by matching subject-specific CT-based bone-plus-implant models to the radiographs using a validated tracking technique. Torque and total work done during abduction were measured using an isokinetic dynamometer. Implant characteristics and surgical techniques that were associated with kinematics/arthrokinematics, strength, or patient-reported outcomes were identified using multiple linear regression. Neck shaft angle, glenosphere size, and retroversion were associated with *in-*vivo kinematics and functional outcomes during abduction after RSA. These findings improve our understanding of how implant design and surgical technique impact kinematics and functional outcomes after RSA. The results highlight the necessity of *in vivo* data to validate cadaver-based research and computer simulations of joint function after RSA, emphasizing that those models do not account for the dynamic healing process and neuromuscular adaptations that occur after surgery.

## 1. Introduction

Reverse shoulder arthroplasty (RSA) is a common procedure used to reduce pain and restore function in patients with glenohumeral osteoarthritis with associated rotator cuff arthropathy [1]. In recent years, the number of primary reverse shoulder arthroplasties has increased by 191 %, with 63,845 RSA’s performed in 2017 [2]. Initially designed to address shortcomings of the anatomic total shoulder arthroplasty, RSA establishes a more medialized center of rotation, increasing stability and longevity of the implant [3]. Recent improvements in prosthesis design have resulted in expanded indications for RSA, including glenohumeral osteoarthritis with attenuated rotator cuff, proximal humerus fractures, inflammatory arthritis, rotator cuff arthropathy, pseudoparalysis, and glenoid bone loss precluding anatomic total shoulder arthroplasty [4-6], that account for its increased popularity. Despite these improvements and favorable patient reported outcomes after RSA, functional outcomes remain suboptimal. Patients continue to experience functional deficits after RSA such as difficulty with toileting and inability to wash their back [7]. Furthermore, RSA is frequently associated with complications, such as scapular notching and limited abduction range of motion (ROM), due to suboptimal implant positioning, which could also limit the postoperative function [8]. Previous computer model and cadaver-based studies suggest greater glenosphere size [9,10], eccentricity [11], and inferior tilt [12] are associated with improvements in abduction ROM, while greater lateralization is associated with improvements in abduction strength [13,14]. Additionally, increasing retroversion angle [9,15, 16] as well as smaller humeral neck shaft angle [17], have been associated with decreased scapular notching and increased ROM. Controversy remains regarding the ideal implant configuration and prosthesis geometry for maximizing shoulder function after RSA [1] due to the lack of quantitative *in vivo* data to guide this debate. Biplanar fluoroscopy is a powerful tool that could improve high quality input data to create in-vivo computational models that are more representative of postoperative shoulder function after RSA. In-vivo analysis after RSA allows for the investigation of arthrokinematics of RSA, defined as the center of contact between the implant liner and the glenosphere, which may provide insight into impingement and wear of the articulating surfaces in the prosthesis.

The aims of this study were to (1) identify surgical techniques (i.e. retroversion and glenosphere tilt) and implant geometry factors (i.e. eccentricity and lateralization) that are associated with *in vivo* kinematics and arthrokinematics after RSA and (2) to identify surgical techniques and implant geometry factors that are associated with better clinical outcomes, including both functional outcomes such as strength and range of motion as well as patient reported outcomes (PROs). Based upon previous computational models and cadaver biomechanical studies [9-18], we hypothesized that a smaller humeral neck shaft angle would be associated with more GH abduction ROM and better clinical outcomes, more humeral retroversion would be associated with a more posterior contact path on the glenosphere, increased abduction ROM, and better clinical outcomes, and more lateralization would be associated with a greater scapulohumeral rhythm (SHR), more abduction strength, and better clinical outcomes.

## 2. Methods

### 2.1. Level of evidence: level iv

A total of 35 patients who received RSA (17M, 18F, 72.8 ± 7.3 years) within the past 2.2 ± 1.1 years consented to participate in this IRB-approved study (Table 1). IRB Approval Number: CR19070432–008. Inclusion criteria were: age 18 to 95 years, patients who received RSA between 2014 and 2020 for rotator cuff arthropathy, irreparable cuff tear, or arthritis. All patients must have completed a supervised postoperative rehabilitation protocol. Pregnant females, in addition to patients who underwent RSA secondary to proximal humerus fracture, revision RSA, ongoing or prior joint infection, or had active axillary nerve of C5 nerve root dysfunction were excluded from this study. The majority of patients in this study received RSA due to primary rotator cuff arthropathy (22) with the second most common indication being osteoarthritis (7). Neither the supraspinatus nor infraspinatus was resected if present, and the subscapularis was repaired in all patients in which it was repairable. RSA was performed by a single surgeon using a standard 135° inlay humeral implant (135°IHI)(Arthrex; Naples, Florida, USA) or a 145° onlay humeral implant (145°OHI)(Tornier, Stryker; Kalamazoo, MI, USA). Twenty five patients received 145° OHI while 9 patients received 135°IHI (Table 1). All patients completed a standardized rehabilitation protocol that included at home and formal physical therapy.

**Fig. 1.**
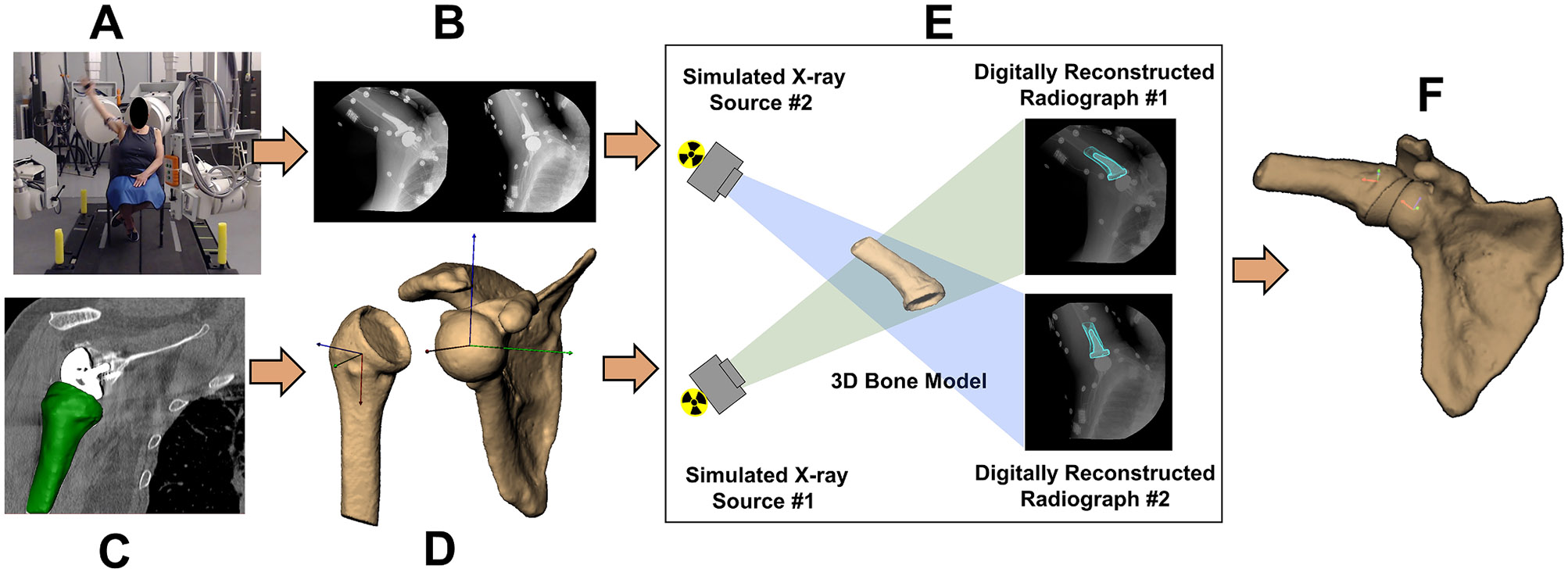
Data Work Flow **(A)** Participants performed abduction in the scapular plane with their affected shoulder while **(B)**synchronized biplane radiographs were collected. **(C)**CT scans of the affected shoulder were collected and **(D)**used to create 3D bone models with anatomical coordinate systems. **(E)**3D glenohumeral positions were determined using a validated CT model-based tracking process. **(F)**6 Degrees of Freedom(DOF) kinematics were calculated.

*In vivo* shoulder kinematics were measured using dynamic biplane radiography. Participants sat upright within a custom biplane radiography system and were instructed to remain still while looking straight ahead with their hands placed on their lap for a single static trial. Participants were then instructed to perform scapular plane abduction throughout their entire pain free ROM such that the participant completed one full ROM cycle (including abduction and adduction) in just under 4 s (Fig. 1A). Uniplanar motion was reinforced by strapping a laser pointer to the participant’s hand and asking them to keep the pointer within a 12-foot-high vertical line of tape. Synchronized biplane radiographs were collected while patients were abducting at 50 images per second for 2 s (focused on the abduction motion) (90 kV, 50 mA, 2 ms pulse duration) and simultaneously conventional motion capture (100 Hz, 12-camera Vicon motion systems, Oxford, UK) was collected to measure shoulder motion during 3 scapular plane abduction trials (Fig. 1B). Over all participants, there were 4 imaging trials that were not of appropriate quality to track due to suboptimal patient positioning. All participants had at least 2 trials analyzed. The maximum radiation exposure during all dynamic trials using the biplane radiography system was estimated to be 1.88mS (estimated using PCXMC, STUK – Radiation and Nuclear Safety Authority, Helsinki, Finland). Biplane radiography data could not be processed from one participant due to their body habitus.

CT scans (0.47 × 0.47 × 0.625 mm voxels) of the affected shoulder and distal humerus were acquired from each participant (GE Lightspeed, Waukesha, WI) and were resliced to generate 0.47 × 0.47 × 0.47 mm cubic voxels to optimize the tracking process described below. The average CT scan exposure was 17.9 ± 2.3mSv. From these CT scans, bone tissue of the humerus and scapula with their respective implants were segmented for each participant using a combination of commercial software (Materialise NV, Leuven, Belgium), and manual segmentation (Fig. 1C). Three-dimensional (3D) models of each scapula-plus-implant and each humerus-plus-implant were created from segmented CT scans [19] (Fig. 1D). Markers were interactively placed on the Trigonum Spinae, Angulus Inferior, and Angulus Acromialis, and the center of the glenosphere on the 3D scapula, as well as the center of the humeral tray, and both medial and lateral distal epicondyles on the humerus to create subject-specific coordinate systems for both the humerus and scapula according to International Society of Biomechanics standards [20].

*In vivo* bone motion was determined with sub-millimeter accuracy by matching digitally reconstructed radiographs generated from the subject-specific CT-based bone-plus-implant models of the humerus and scapula to the biplane radiographs using a validated volumetric tracking technique [21] (Fig. 1E) with overall dynamic accuracy in any one direction <0.385 mm for the scapula and <0.374 mm for the humerus [21]. Finally, six degree of freedom GH kinematics were calculated (Fig. 1F).

The kinematic outcomes included GH plane of elevation, GH abduction, scapular upward rotation, and SHR. GH plane of elevation, abduction, and internal/external rotation were calculated using projection angles and described in terms of spherical rotations using custom Matlab code [22]. Scapular upward rotation was calculated using projection angles of the scapula relative to the thorax, with thorax motion defined by reflective markers that were placed on the manubrium, xiphoid process, C7, T10, and opposite acromion process and tracked using conventional motion capture (Vicon Vantage, 12-camera system, 100 Hz). GH abduction and scapular upward rotation were averaged across trials at corresponding humerothoracic abduction angles. SHR was calculated as the ratio of the GH abduction to scapulothoracic motion over each 5° increment of humeral-thoracic abduction and averaged across trials.

3D CAD models of the polyethylene inserts, provided by the manufacturers, were fit into the CT-based humeral tray, allowing for increased precision of our kinematic measurements. The movement of the geometric center of the contacting surface of the polyethylene humeral liner relative to the glenosphere was calculated for every instant GH kinematics were calculated. The location of the center of the polyethylene liner was projected onto the glenosphere throughout the entire abduction motion, and this center of contact location in the superior/inferior (SI) and the anterior/posterior (AP) directions was averaged across trials at corresponding GH abduction angles and normalized to glenosphere size.

Implant geometry parameters (glenosphere eccentricity, neck shaft angle/implant type, glenosphere size), were recorded from surgical notes while surgical techniques (humeral retroversion, glenosphere tilt) were measured on 3D bone models or recorded from surgical notes (glenoid lateralization) (Table 1). Glenoid tilt, humeral retroversion, glenosphere size, lateralization, glenosphere tilt, glenoid eccentricity (a glenosphere implant with a more inferior central axis of rotation), neck-shaft angle/implant type, and retroversion were assessed to see how they affect SHR, the center of contact between the polyethylene and the glenosphere, maximum glenohumeral abduction, and scapular upward rotation.

Clinical outcomes measured include isokinetic torque, peak torque, total work done, and clinical abduction ROM. An isokinetic dynamometer (Biodex) set to 30° per second was used to measure isokinetic torque throughout full ROM abduction and adduction during movement trials performed after the biplane radiography imaging. Each participant performed trials at 50 %, 75 %, and 100 % maximum effort. Participants were instructed to approximate the percentage of effort relative to their perceived maximum effort to correspond with each trial. Peak torque and total work done (the area under the torque versus humerothoracic angle curve) during the 100 % maximum effort trial were normalized to bodyweight and used to characterize strength for both abduction and adduction. Analyzing mechanical joint work allows us to obtain a more comprehensive functional status as it accounts for both torque and range of motion. Lastly, clinical abduction ROM and maximum isometric force at 90° of abduction in the scapular plane were measured using a protractor and a handheld dynamometer, respectively, at the time of testing. The following patient reported outcomes (PROs) were collected at the time of testing on a tablet using standardized assessment forms including, the Constant Murley Score (CMS), American Shoulder and Elbow Surgeons (ASES) Shoulder Assessment, and Disabilities of the Arm, Shoulder, and Hand (DASH).

Implant characteristics and surgical techniques that were associated with the primary kinematics (maximum GH abduction, maximum scapular upward rotation, and SHR) and arthrokinematics (peak superior contact point location, average AP contact path location) were identified by including all subjects in a multiple linear regression using forward selection with SPSS 29.0 software. Associations between kinematics/arthrokinematics and clinical outcomes (PROs, clinical ROM, and isokinetic strength) were evaluated with Pearson’s correlations. Significance was set at *p* < 0.05 for all tests.

## 3. Results

A total of 34 static trials and 98 dynamic movement trials from 34 individuals were included in this analysis.

### 3.1. Associations between implant parameters/surgical technique and kinematics/arthrokinematics

**Fig. 2.**
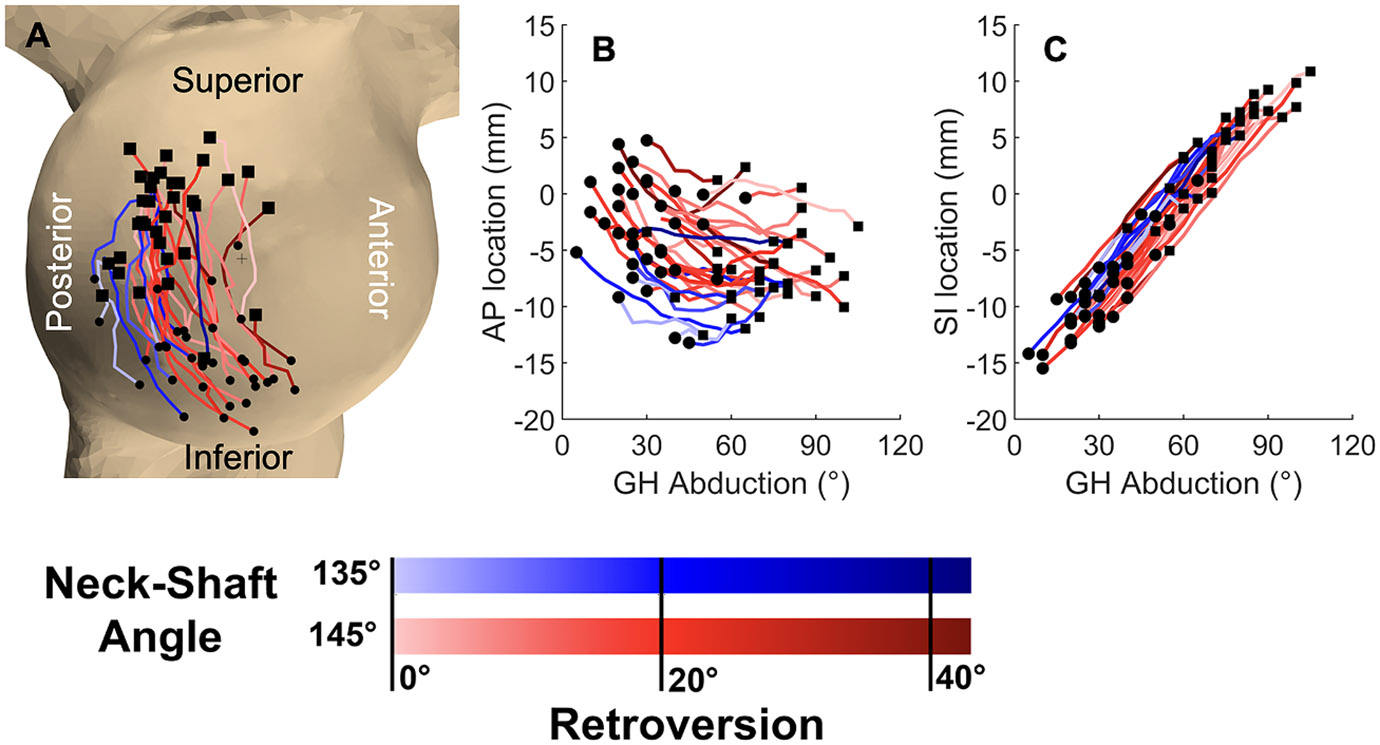
The Effect of Neck Shaft Angle and Retroversion on Contact Path Location. Blue lines represent participants with 135°Inlay humeral implant (IHI) while red lines represent participants with 145°Onlay humeral implant(OHI). Circles indicate the start of abduction and boxes indicate maximum abduction. Darker colors indicate more retroversion and lighter colors indicate less retroversion. **(A)** The path from lower abduction angles (circles) to maximum abduction angles mapped onto the glenosphere for each participant. **(B)** The change in anterior/posterior (AP) location of the contact center with increasing abduction. **(C)** The change in superior/inferior(SI) location of the contact center with increasing abduction.

Greater neck shaft angles and higher retroversion angles were associated with a more anterior contact path (Fig. 2A and B, *R* = 0.778, *p* < 0.001). More specifically, the 145°OHI was associated with a contact path 3.4 mm more anterior than the 135°IHI (Fig. 2A and B, β=0.336). For every 10° increase in retroversion, the contact path was 0.8 mm more anterior (Fig. 2A and B, β=0.079). No associations were found between implant characteristics or surgical techniques and SI contact path location (Fig. 2C). Average SHR was 0.9 ± 0.3. Average maximum scapular upward rotation was 51.8° ±10.3°. Average maximum glenohumeral abduction was 73.8°±16.5°. No associations were found between implant characteristics or surgical techniques and SHR, maximum scapular upward rotation, or maximum glenohumeral abduction (all *p* > 0.050, Table 3).

### 3.2. Associations between kinematics/arthrokinematics and patient reported/ functional outcomes

Average PROs were 73.1 ± 10.9 (range 46.6 to 95.9) for the CMS, 81.8 ± 16.4 (range 35 to 100) for the ASES, and 18.0 ± 15.3 (range 0.0 to 61.7) for the DASH. The ability to perform more work in both abduction and adduction was associated with higher peak superior contact points (*R* = 0.554, *p* < 0.001 and *R* = 0.453, *p* = 0.007, respectively) and greater maximum GH abduction (*R* = 0.465, *p* = 0.006, and *R* = 0.422, *p* = 0.013, respectively). Greater torque during abduction was associated with a more superior contact path (*r* = 0.365, *p* = 0.034). No other associations between clinical variables and kinematics or PROs were found (all *p* > 0.050, Table 3).

### 3.3. Associations between implant parameters/surgical technique and clinical outcomes

Larger glenosphere size was associated with the ability to produce greater peak torque in abduction (*R* = 0.631, *p* < 0.001) and adduction (*R* = 0.558, *p* < 0.001) and to perform more work in both abduction (*R* = 0.444, *p* = 0.008) and adduction (*R* = 0.478, *p* = 0.004). Furthermore, greater retroversion was associated with greater peak torque in both abduction (*R* = 0.420, *p* = 0.014) and adduction (*R* = 0.447, *p* = 0.008). Additionally, more eccentricity was associated with more work (*R* = 0.335, *p* = 0.053) and torque (*R* = 0.393, *p* = 0.022) during adduction. Lastly, a greater neck shaft angle was associated with more abduction ROM (*R* = 0.358, *p* = 0.037)(Table 2). No associations between implant parameters or surgical technique and PROs were found (all *p* > 0.050, Table 3).

## 4. Discussion

The main findings of this study were that neck shaft angle, retroversion, and glenosphere size were associated with kinematics and functional outcomes after RSA; however, contrary to our hypotheses, no associations were found between surgical technique or implant geometry and PROs.

The data did not support our hypothesis that a smaller neck shaft angle would be related to PROs. The inability to identify these associations could be due to several factors, including the relatively small sample size, patient expectations that may confound the relationship between function and patient-reported satisfaction, or it is possible that there is no association between neck shaft angle and PROs.

Contrary to our hypothesis, greater neck shaft angles and higher retroversion angles were associated with a more anterior contact path between the polyethylene insert and the glenosphere. It is possible that increased retroversion causes the humeral component to sit more anteriorly on the glenoid when at rest, which could account for the more anterior contact path throughout the abduction motion. The in vivo relationships between contact path and implant parameters reported here provide novel information to improve our understanding of implant wear. Despite knowing that volumetric wear of the polyethylene is high in RSA, there has been little information about the abrasive wear caused by the sliding of the articular surfaces of reverse implants that could be affected by contact path location [23]. These results suggest that future studies may focus on how implant design may affect anterior/posterior wear patterns.

Greater glenosphere size, greater retroversion, and greater eccentricity were all associated with greater strength, and a greater neck shaft angle was associated with greater clinical abduction ROM. This is in line with our hypothesis that greater retroversion would be associated with improved clinical outcomes. These results are fairly similar to Muller et al. who also found that increased glenosphere size lead to increased abduction strength after RSA [24]. While the strength measurements were normalized to body weight, it may have not completely removed the bias of males being more likely to receive a larger glenosphere, which may confound the results. Our finding that greater retroversion is associated with more abduction strength contradicts previous reports. Multiple simulation studies, including Henninger et al. and Gulotta et al., did not report any significant effects of retroversion on abduction strength after RSA [25,26]. Whereas computer simulations are widely accepted as a reliable and accurate, they do bear some limitations in assessing the active and dynamic changes that may occur *in vivo*. It is possible that the simulations could be improved to replicate the *in vivo* condition after RSA more closely. Our finding that greater neck shaft angle is associated with better clinical ROM during abduction agrees with previous literature that demonstrated smaller neck shaft angles were associated with less abduction ROM [27,28]. A proposed explanation for this finding is that smaller humeral neck shaft angles could lead to excessive proximalization, in turn causing an earlier impingement of the greater tuberosity on the acromion [29]. Our finding that increased strength was associated with greater abduction ROM and a more superior contact path are in agreement with previous literature as Alta et al. clinically deduced that impaired shoulder strength is a likely cause of active ROM limitations after RSA [30]. While this aligns with previous literature, a greater abduction angle does not necessarily imply greater peak torque as patients may regain a high range of motion after RSA but they possess only low or moderate strength. The novelty of our findings remains, as these are in-vivo measurements of shoulder kinematics under physiologic loading that account for healing and neuromuscular changes after RSA.

Contrary to our hypothesis and previous cadaveric studies [13], we were unable to find any *in vivo* evidence that lateralization impacts strength. Additional analysis is needed to determine if the relationship between lateralization and strength is more complicated *in vivo*, and potentially confounded by the difference between post-surgical and native lateralization. Furthermore, we were unable to support our hypothesis that greater lateralization would result in greater SHR. The results suggest that the relationship between scapulothoracic motion and GH motion is altered after RSA and the relationship is not affected by lateralization.

Strengths of this study include the precise *in vivo* kinematics data collected, the collection of multiple trials of the same movement to increase the likelihood that the kinematics reflect the typical movement pattern [31], and a wide array of outcomes assessed (kinematics, arthrokinematics, strength, clinical ROM, PROs). Limitations of this study include the small sample size and some of the device design options were not evenly distributed (e.g. neck shaft angle) among patients. Only one planar movement was analyzed, therefore additional relationships may be observed when performing other functionally relevant shoulder movements. Additionally, since there were no previous *in vivo* data on kinematics/arthrokinematics of patients after RSA and we did not want to overlook potentially significant relationships, the statistical tests did not control for multiple comparisons. The results of this study can be used in the future to design a more rigorous study that focuses on the most relevant implant design factors and surgical techniques identified in this study. An additional limitation is that strength, range of motion, and kinematics of the contralateral shoulder were not considered. More data is needed so that comparisons can be made between the shoulder kinematics and strength after RSA and the contralateral side of each patient and/or a group of matched controls.

This study is one of the first to examine the complex *in vivo* relationship between surgical technique, implant geometry, functional outcomes, and PROs after RSA. The findings suggest that implant parameters and surgical techniques are associated with kinematic outcomes and functional performance following RSA, while PROs may be influenced by a different set of factors. Studies of this nature are important to provide insight into the mechanisms affecting functional outcomes and highlight the importance of having *in vivo* data to confirm cadaver-based research and computer simulations that do not account for healing and changes in neuromuscular control after surgery. This study takes a significant step in bridging the gap between theoretical models and real-world outcomes. It underlies the necessity of *in vivo* data to validate cadaver-based research and computer simulations emphasizing that these models do not encompass the dynamic healing process and neuromuscular adaptations that occur after surgery. This study serves as a vital building block for future research endeavors and paves the way for more refined approaches to enhance the outcomes of RSA.

## Figures and Tables

**Table 1:** Patient Demographics and Implant Parameters: Summary of patient demographics, the implant parameters and surgical techniques used in this study.

Age (Years)	Sex	Time from surgery (Years)	Surgical Indication		Neck Shaft Angle		Eccentricity (# of Subjects)	Glenosphere Size				Lateralization (mm)	Retroverion (Degrees)
			Rotator cuff arthropathy (# of Subjects)	Osteoarthritis (# of Subjects)	135° (# of Subjects)	145° (# of Subjects)		33 mm (# of Subjects)	36 mm (# of Subjects)	39 mm (# of Subjects)	42 mm (# of Subjects)		
72.8 ± 7.3 Range (56 to 88)	17M 18F	2.2 ± 1.1	22	7	9	25	7	1	20	7	6	2.6 ± 2.8 Range (0–10)	15.9 ± 11.2 Range (0.6 - 42.8)

**Table 2:** Average peak torque (Newton meters per kilogram) and total work done (joules) during abduction and adduction using an isokinetic dynamometer (Biodex).

Peak Torque		Total Work Done	
Average Abduction (Nm/ kg)	Average Adduction (Nm/ kg)	Average Abduction (Joules)	Average Adduction (Joules)
0.38 ± 0.14	0.35 ± 0.20	0.59 ± 0.31	0.50 ± 0.38

**Table 3:** Statistical results for associations between implant parameters, surgical technique variables, kinematic measurements, and PROs.

*Associations between* *implant parameters/* *surgical technique and* *Kinematics/* *Arthrokinematics*	Associations Between Kinematics/Arthrokinematics and Patient Reported/ Functional Outcomes			*Associations Between Implant Parameters/Surgical Technique and Clinical Outcomes*				
Greater neck shaft angles and higher retroversion angles were associated with a more anterior contact path	More work in abduction and adduction was associated with...		Greater torque during abduction was associated with a more superior contact path	Larger glenosphere size was associated with...		Greater retroversion was associated with greater peak torque in both abduction and adduction	More eccentricity was associated with more work and torque during adduction	Greater neck shaft angle was associated with more abduction ROM.
	Higher peak superior contact points	Greater Maximum GH abduction		Greater peak torque in abduction and adduction	More work in both abduction and adduction			
*R* = 0.778, *p* < 0.001	Abduction: *R* = 0.554, *p* < 0.001 Adduction: *R* = 0.453, *p* = 0.007	Abduction: *R* = 0.465, *p* = 0.006 Adduction: *R* = 0.422, *p* = 0.013	*R* = 0.365, *p* = 0.034	Abduction: *R* = 0.631, *p* < 0.001 Adduction: *R* = 0.558, *p* < 0.001	Abduction: *R* = 0.444, *p* = 0.008 Adduction: *R* = 0.478, *p* = 0.004	Abduction: *R* = 0.420, *p* = 0.014 Adduction: *R* = 0.447, *p* = 0.008	Work: (*R* = 0.335, *p* = 0.053) Torque: (*R* = 0.393, *p* = 0.022)	*R* = 0.358, *p* = 0.037
